# Hybrid Natural Fiber Composites of Polylactic Acid Reinforced with Sisal and Coir Fibers

**DOI:** 10.3390/polym17010064

**Published:** 2024-12-30

**Authors:** Wipoo Sriseubsai, Ariya Praemettha

**Affiliations:** Department of Industrial Engineering, School of Engineering, King Mongkut’s Institute of Technology Ladkrabang, Bangkok 10520, Thailand

**Keywords:** sisal, coir, PLA, mixture design, mechanical properties

## Abstract

This study explored the tensile and impact strength of polylactic acid (PLA) through the incorporation of sisal and coir fibers. Hybrid natural fiber composites were prepared using PLA as the matrix and sisal and coir fibers as the reinforcements. The hybrid composites were prepared with an internal mixer, followed by compression molding. A constrained mixture design was employed to determine the optimal material combinations and their effects on the tensile and impact strength. Confirmatory experiments based on response surface methodology revealed no significant differences in the data means at the 0.05 significance level. PLA reinforced with sisal fibers alone exhibited the highest tensile strength of 75.36 MPa but demonstrated a low impact resistance of 12.94 kJ/m^2^ at a 95.22:4.78 (PLA:sisal by volume) ratio. Conversely, the maximum impact resistance of 36.71 kJ/m^2^ was achieved with PLA and coir at the same ratio. An optimal blend, consisting of 95.22% PLA, 0.78% sisal, and 4.0% coir by volume, resulted in a tensile strength of 51.08 MPa and an impact strength of 26.59 kJ/m^2^, outperforming other mixtures and pure PLA in the mechanical properties. Additionally, water absorption tests showed that reinforcement with sisal and coir fibers increased both water absorption and stability over 60 h.

## 1. Introduction

Many researchers have attempted to enhance the mechanical properties of biodegradable plastics derived from natural resources, such as polylactic acid (PLA) and natural polyethylene (NPE). However, PLA exhibits significant drawbacks, such as limited commercial availability, poor processability, low toughness, inadequate moisture stability, and high production costs [1,2]. Studies have shown that PLA can serve effectively as a composite matrix when reinforced with fibers [3,4,5,6,7,8,9,10,11,12,13,14]. Compared to synthetic fibers, natural fibers offer notable advantages, including low weight, biodegradability, and superior strength and stiffness. Previous studies have predominantly focused on single natural fiber-reinforced composites, such as flax [1], sisal [15], pineapple leaves [16], coir [17], hemp [18], cotton [19], etc. Caloline et al. developed PP/EPDM composites reinforced with sisal fibers [20], reporting enhancements in the tensile and flexural properties. Bravo et al. [21] studied bio-composites reinforced with short birch fibers, using NPE as the matrix. The study also included comparisons with two alternative matrices: linear low-density polyethylene (LLDPE) and high-density polyethylene. The birch fiber content was varied at 10, 20, 30, and 40 wt%, with a coupling agent incorporated to enhance performance. The results demonstrated that the tensile strength increased across all matrices with the fiber addition, with LLDPE exhibiting the highest tensile strength. Acoustic emission testing indicated that the coupling agent improved the mechanical properties of all matrices. In another study, Cheng et al. [7] investigated the mechanical and thermal properties of PLA composites reinforced with chicken feathers in varying amounts (2–10 wt%). The tensile strength of the PLA composite reinforced with chicken feathers improved with the increasing feather content, reaching a maximum at 5%, which represented a 16% improvement compared to pure PLA. Scanning electron microscopy (SEM) analysis revealed a uniform dispersion of feathers within the matrix. Analysis of the dynamic mechanical properties, mechanical properties, and thermal stability indicated an increased modulus, a slight reduction in the glass transition temperature (*T*_g_), and improved thermal stability relative to PLA. Furthermore, Oksman et al. [1] studied the mechanical properties of PLA composites reinforced with flax fibers at 30 wt% and 40 wt%. It revealed that the tensile strength was highest at a 30 wt% flax fiber content but decreased with a further increase in the fiber content. The findings were compared to polypropylene composites reinforced with flax fibers at equivalent fiber contents, given polypropylene’s widespread use in industry. Both composite types exhibited similar tensile strengths. In the study by Bax and Mussig [22], the performance of PLA composites reinforced with Cordenka and flax fibers was compared, with a focus on the tensile strength and impact resistance at fiber contents of 10–30 wt%. Their findings revealed that the tensile strength improved significantly when 30 wt% Cordenka fibers were used, while the modulus of the material increased with the addition of flax fibers. SEM analysis revealed relatively low adhesion between the matrix and both fiber types.

Currently, reports on hybrid composites, which combine different kinds of natural fiber-reinforced composites, are increasingly common. These composites are formed by incorporating multiple fiber types into a single matrix, offering distinct advantages over using a single natural fiber. Notably, the unique properties of each type contribute to enhanced composite performance, especially in terms of the mechanical properties. For example, studies on sisal and banana fiber-reinforced epoxy hybrid composites show improved mechanical strength and reduced water absorption [23]. Yusoff et al. [24] focused on PLA composites reinforced with kenaf, bamboo, and coir fibers to evaluate their impact on the mechanical properties. The findings indicated that the addition of natural fibers increased the tensile strength of the material, with those containing jute, bamboo, and coconut fibers showing up to a 78% increase compared to that of plain PLA. The study further highlighted that jute and coconut fibers improved the tensile strengths and moduli, while jute and bamboo fibers contributed to an increased modulus, and coconut fibers enhanced the toughness. Therefore, combining different reinforcement fibers yielded better mechanical properties than using a single fiber type. Meanwhile, Shanmugam et al. [25] examined the deformation and mechanical properties of polyester composites reinforced with palm and jute fibers at various fiber ratios (100:0, 75:25, 50:50, 25:75, and 0:100). The results showed that the addition of both fiber types enhanced the tensile and compressive strengths compared to composites reinforced with only one type of fiber. While increasing the proportion of jute fibers improved the tensile strength, it decreased when the ratio reached 100% jute. In addition, the impact resistance was lower in composites containing both types of fibers. Material stability analysis demonstrated no significant change in the stability of the composites with palm and jute fibers.

Meanwhile, the mixture ratio of natural fiber hybrid composites is crucial for achieving the optimal properties, yet it remains unexplored. Mixture design experiments are typically used in response surface studies, where the product under analysis comprises multiple components, and the proportion of each is independent of the others. Importantly, the proportions of these components are constrained to sum to 1 (or 100%). These techniques primarily aim to optimize mixtures whose properties depend on the relative proportions of their constituents. For example, a previous study employed a mixture design to determine the optimal proportion for the paste component in bonded roller-compacted fiber-reinforced polymer-modified concrete [26].

In this work, hybrid composites were developed using compression molding, incorporating short sisal fibers, known for their high tensile strength, and coir fibers, valued for their high elongation properties within a PLA matrix. The effects of the sisal and coir fiber contents on the mechanical properties and water absorption behavior of the hybrid composites were investigated. Furthermore, the proportions among the components of the composites were optimized using a constrained mixture design method, which is well suited for identifying mixtures of dependent parameters.

## 2. Materials and Methods

### 2.1. Materials

#### 2.1.1. Polymer PLA

PLA 4043D from NatureWorks, supplied by BC POLYMER MARKETING, Bangkok, Thailand served as the matrix of the composite material. Its physical properties are detailed in Table 1.

#### 2.1.2. Reinforcement Fibers

Sisal fibers, cultivated in Phetchaburi province, Thailand, exhibited an average diameter of 127 µm and a density of 1.16 g/cm^3^. Coir fibers, sourced from TT&G Fiber Co., Ltd., Khlong Toei, Thailand, had an average diameter of 213 µm and a density of 1.39 g/cm^3^. Both fiber types were chemically treated and subsequently oven-dried at 60 °C for 24 h. The as-prepared fibers were then cut to a uniform length of 3 mm.

### 2.2. Methodology

#### 2.2.1. Measurement of the Properties of the Reinforcement Fibers

The cross-sectional area of the fibers was observed using a digital microscope (Axiolab, ZEISS, Oberkochen, Germany). The area was estimated from captured images and analyzed using SemAfore 5.21 software. Fiber density was measured with an electronic densimeter (BSA series, Scientific Promotion Co., Ltd. Bangkok, Thailand) following the Archimedes method, as suggested by Amiri et al. [33], and in accordance with ASTM D8171 [34].

For density measurements, the weight of the fiber in the air (*M_air_*) was recorded. Subsequently, the fiber was submerged in a fluid using a weighing basket, and the submerged weight (*M_submerged_*) was recorded. The densities of the fluid (*ρ_fluid_*) and fibers (*ρ_fiber_*) were calculated using the following formula [33]:

(1) $$\mathrm{Density}\;\mathrm{of}\;\mathrm{fiber}\;(\rho_{fiber})=\frac{M_{air}}{M_{air}-M_{submerged}}\times \rho_{fluid}$$

#### 2.2.2. Reinforcement Fiber Tensile Test

Single-fiber tensile tests were performed following ASTM D3379 [35]. The tests utilized a universal testing machine (A6-X, SHIMADZU at KMITL, Bangkok, Thailand) equipped with a 10 kN load cell, with a gauge length of 25 mm and a crosshead speed of 1 mm/min. A total of 25 samples were tested, prepared, and mounted with paper.

#### 2.2.3. Experimental Design and Statistical Analysis

**Figure 1:**
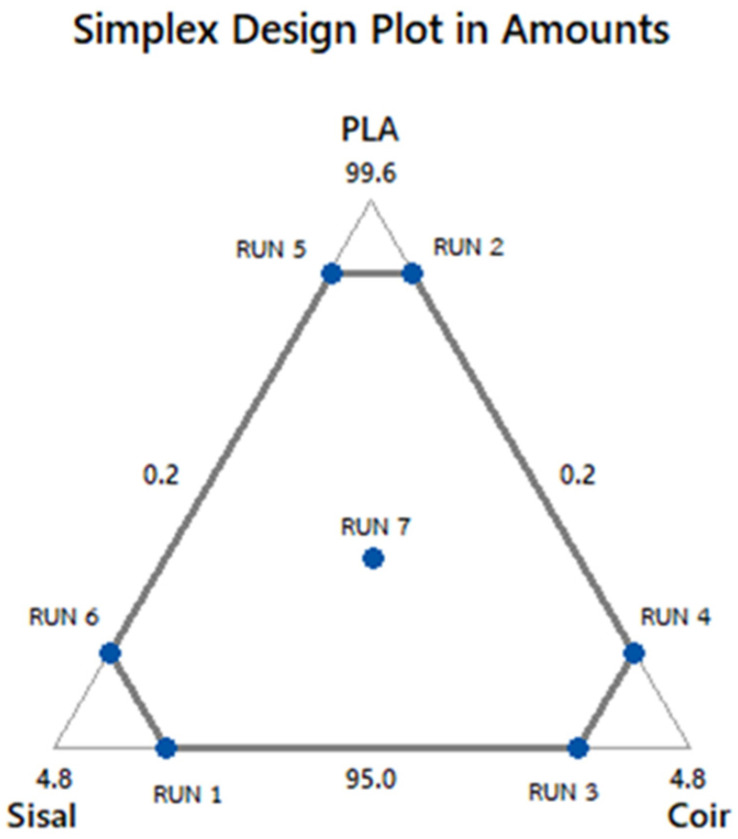
The constrained mixture design plot in proportions of PLA, sisal, and coir.

In this work, a constrained mixture design was employed to evaluate the effects of the amount of PLA (X_1_,%), sisal (X_2_,%), and coir (X_3_,%) by volume on the response, tensile strength, impact strength, and water absorption. The mixture constraint ensured that the total percentage of the components equals 100%. Table 2 details the factors and levels of the design, while the seven experimental runs, generated using Minitab v.17 software, are presented in Figure 1. Furthermore, analysis of variance (ANOVA) and statistical analysis were performed via Minitab v.17.

#### 2.2.4. Composite Preparation

PLA and short fibers were dried in an oven at 60 °C for 6 h. The composites were then compounded via an internal mixer (MX75, Chareon TNT Co., Ltd. Bangkok, Thailand) at 190 °C for 5 min at 40 rpm, followed by cutting into pieces using a plastic crusher machine. The resulting composite pellets were dried in an oven at 100 °C for 2 h. The samples of the composites were formed with a compression molding machine (PR2D-W300L350-PM-WCL-HMI, Chareon TNT Co., Ltd., Bangkok, Thailand). The hot-pressing process involved preheating at 190 °C and 2 MPa for 1 min, followed by compression at 3.44 MPa for 3 min at the same temperature and cooling under pressure for 10 min.

#### 2.2.5. Mechanical Testing

Tensile testing was conducted in accordance with ASTM D638 [36] Type I (length 165 mmand 7 mm thickness) using a universal testing machine (A6-X, SHIMADZU) equipped with an extensometer. The crosshead speed was set to 50 mm/min, and testing was performed at room temperature on five samples. The Izod impact strength was measured following ASTM D256 [31] using an impact machine (QC-639G, COMETECH Testing Machine, Bangkok, Thailand), with five samples tested. The dimensions of the specimens were 55 × 13 × 1 mm. In addition, water absorption was evaluated according to ASTM D570 [37] by immersing dried samples in distilled water at 25 °C for 24 h, with five samples tested. Water absorption of the composites was determined as described in the following equation [38]:

(2) $$\mathrm{Water}\;\mathrm{absorption}=\frac{\mathrm{Wet}\;\mathrm{weight}\;-\;\mathrm{Dry}\;\mathrm{weight}}{\mathrm{Dry}\;\mathrm{weight}}\times 100$$

## 3. Results

### 3.1. Physical Properties of the Reinforcement Fibers

**Figure 2:**
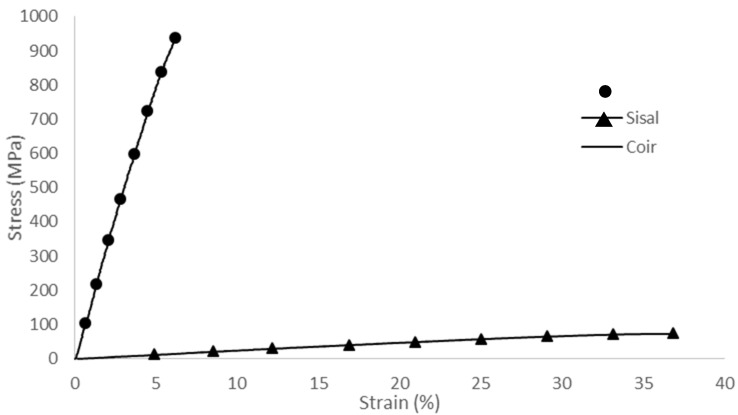
Stress–strain of sisal and coir fibers.

The mechanical properties of a single fiber were tested following ASTM D3379 [35]. The sisal fibers exhibited a tensile strength of 765–1095 MPa, elongation at break of 5.5–6.6%, and Young’s modulus of 136–170 MPa. In contrast, the coir fibers showed a tensile strength of 54–80 MPa, elongation at break of 27–43%, and Young’s modulus of 2–3 MPa. These results are shown in Figure 2. The sisal fibers demonstrated a high specific strength, while the coir fibers exhibited high elongation, making both suitable for use as reinforcing materials in polymeric resin matrices for structural composite applications.

**Figure 3:**
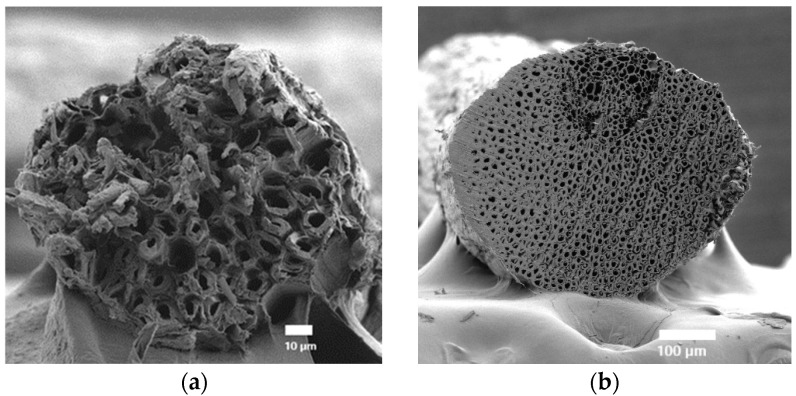
Cross-sectional view of (**a**) sisal and (**b**) coir fibers.

SEM micrographs illustrate the densified and porous structures of the sisal and coir fibers, as shown in Figure 3. The cross-section of the sisal fibers revealed multiple elliptical lumens and thin cell walls, whereas the coir fibers displayed a uniform small lumen and a thick cell wall. The irregular “cuticle” layer on the fiber surfaces enhanced adhesion with the matrix material. Analysis of the fiber diameters using SemAfore 5.21 software, as detailed in Table 3, indicated that the coir fibers exhibited a larger diameter compared to the sisal fibers.

The density of the sisal and coir fibers was tested using the Archimedes method, as shown in Table 4. The results showed that the sisal had less density than the coir fibers.

### 3.2. Statistical Analysis of the Models

**Figure 4:**
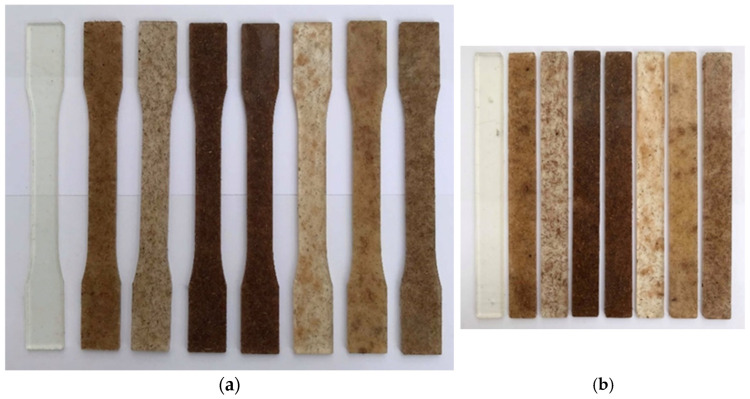
Sample preparation for mechanical properties: (**a**) tensile strength and (**b**) impact strength.

**Figure 5:**
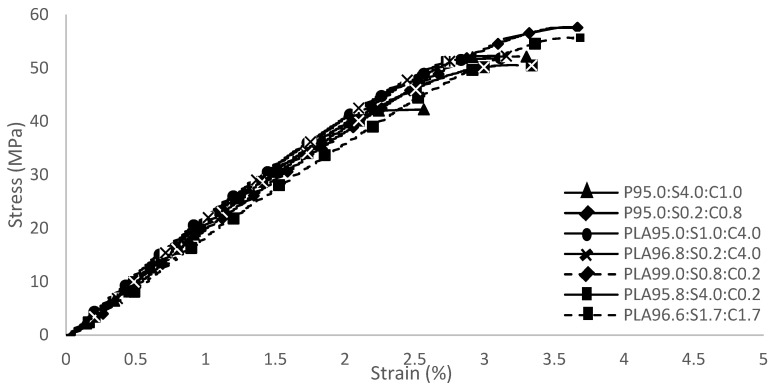
Stress curve of the composites.

The specimens for the tensile and impact strengths were prepared following ASTM D638 [36] and D256 [31] standards, respectively (Figure 4). The experimental results of the tensile and impact strengths of the seven mixtures are presented in Figure 5 and Table 5. All data were fitted to quadratic models, with the ANOVA results provided in Table 6 and Table 7. The regression model for each response exhibited a *p*-value below 0.05, indicating the high significance of the quadratic model. Table 8 shows the elongation and modulus of each mixture, showing that the composite containing sisal and coir displayed increased elongation and modulus, enhancing energy absorption with a higher impact strength.

### 3.3. Tensile Strength of the Hybrid Composite

**Figure 6:**
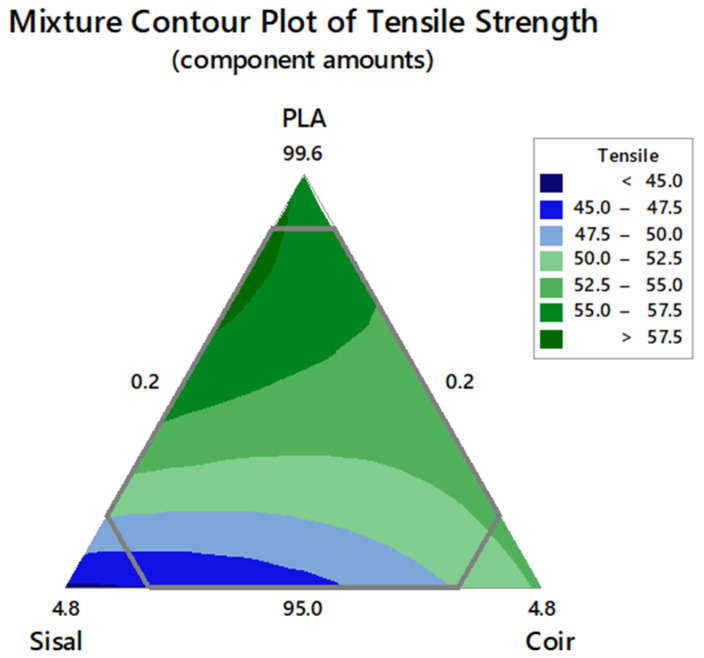
Mixture contour plot of the tensile strength.

The mixture contour plot for the tensile strength revealed that the sisal and coir fibers had a minimal influence on the tensile strength compared to PLA, as shown in Figure 6. A gradual decline in the tensile strength was observed with the increasing sisal and coir fiber contents, likely due to poor interfacial adhesion between the polymer matrix and these fibers. This phenomenon is generally observed in composites with mismatched hydrophobicity of the polymer matrix and hydrophilicity of sisal and coir fibers [39]. The hybrid composites achieved a maximum tensile strength of 58.09 MPa with a PLA:sisal:coir (% by volume) of 98.58:1.22:0.20, meeting the composite desirability of 1 and falling within the acceptable range.

### 3.4. Impact Strength of Hybrid Composites

**Figure 7:**
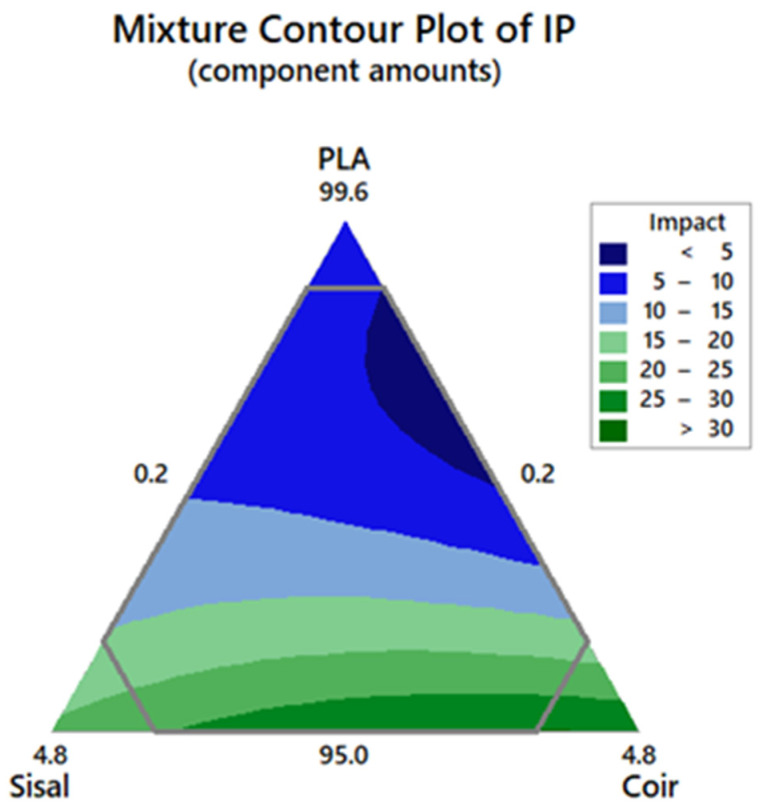
Mixture contour plot of the impact strength.

In terms of the impact strength, the sisal and coir fibers showed a significant effect on the impact strength, as depicted in Figure 7. These findings suggest that increasing the sisal and coir fiber contents in the mixture improved the impact strength of the hybrid composites. The relative impact strength is enhanced with respect to the matrix. The highest impact strength, recorded at 30.14 kJ/m^2^, was achieved with a PLA:sisal:coir ratio of 95.00:1.08:3.92.

### 3.5. Optimization of Hybrid Composites

**Figure 8:**
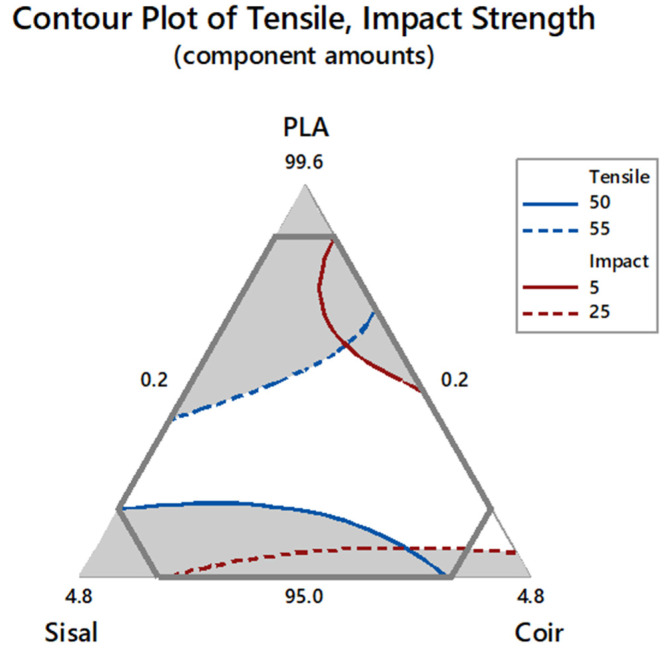
Overlaid contour plot of the tensile and impact strengths.

The overlaid contour plot of the tensile strength and impact strengths is presented in Figure 8. The hybrid composite attained the highest tensile strength of 51.08 MPa and impact strength of 26.59 kJ/m^2^ at a PLA:sisal:coir ratio of 95.22:0.78:4.00.

### 3.6. Test Results Confirmation

Using the overlaid response optimizer, the optimal composition for a hybrid composite material reinforced with sisal and coir fibers was found to consist of 95.22% PLA, 0.78% sisal, and 4.00% coir. This statistical tool identifies the ideal conditions by overlaying the desirability functions for multiple responses to achieve a balanced, overall best solution. To validate the predicted results, a *t*-test was performed, comparing the tensile and impact strength values obtained from the optimizer’s predictions with those measured through experimental testing.

The results presented in Table 9 show that the *p*-values for both the tensile and impact strengths were less than 0.05. In statistical hypothesis testing, a *p*-value represents the probability of obtaining the results as extreme as, or more extreme than, the observed data under the assumption that the null hypothesis is true. A *p*-value below 0.05 indicates that the differences between the predicted and observed values are statistically significant. Therefore, the results confirm that the predictions generated by the overlaid response optimizer align closely with the actual testing data, demonstrating the reliability of the optimizer’s output.

### 3.7. Comparison of Reinforcement with Dual Natural Fibers and Single Natural Fiber in PLA-Based Composites

**Figure 9:**
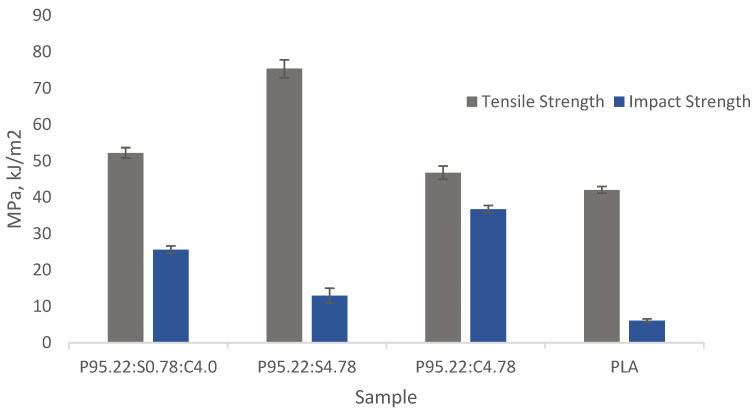
Tensile strength and impact resistance of P95.22:S0.78:C4.0; P95.22:S4.78; P95.22:C4.78; and PLA.

The tensile strength and impact resistance of PLA-based composites reinforced with a hybrid of sisal and coir fibers (P95.22:S0.78:C4.0) were evaluated against PLA composites reinforced solely with sisal fibers (P95.22:S4.78) or coir fibers (P95.22:C4.78). The fiber reinforcement ratios in the single-fiber composites matched the total fiber content of the dual-fiber composite, as determined by the overlaid response optimizer analysis, with a reinforcement rate of 4.78% (Figure 9).

The optimal mixing ratio determined using the overlaid response optimizer demonstrates the complementary reinforcement provided by two types of fibers in achieving the desirable mechanical properties. For instance, PLA reinforced with sisal fibers exhibited the highest tensile strength at 75.36 MPa but a low impact resistance of 12.94 kJ/m^2^. This indicates that the increased amount of sisal fibers enhances the tensile strength due to improved adhesion with the PLA matrix, enabling better stress transfer and distribution. However, the impact resistance showed minimal improvement at this fiber concentration. Conversely, PLA reinforced with coir fibers demonstrated the highest impact resistance of 36.71 kJ/m^2^ but a relatively low tensile strength of 46.78 MPa. This confirms that reinforcing PLA with a single fiber type results in a noticeable enhancement in only a specific mechanical property. Sisal fiber reinforcement significantly increased the tensile strength but had a minimal effect on the impact resistance, while coir fiber reinforcement enhanced the impact resistance without substantially increasing the tensile strength. In contrast, reinforcing PLA with both sisal and coir fibers synergistically improved its mechanical properties. The hybrid reinforcement yielded a higher tensile strength and impact resistance compared to using a single fiber type, demonstrating the complementary benefits of these natural fibers.

### 3.8. Water Absorption of the Hybrid Composites

**Figure 10:**
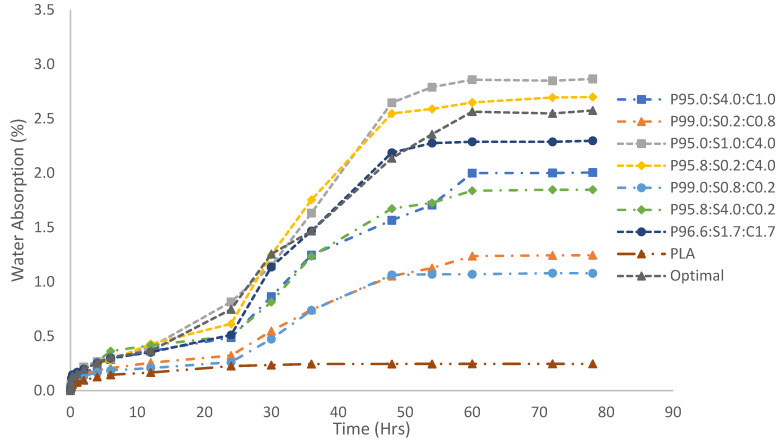
Water absorption of hybrid composites.

The water absorption behavior of the hybrid composites is presented in Figure 10. The results showed that PLA exhibited stabilization in water absorption after 24 h of testing, reaching a maximum of 0.25%. In contrast, all composite materials, regardless of their mixing ratios, stabilized after 60 h, with water absorption values exceeding those of PLA. The composite from RUN 3, with a composition of 95.00% PLA, 1.0% sisal fibers, and 4.0% coir fibers, exhibited the highest water absorption at 2.87%. Similarly, the composite in RUN 4, comprising 95.8% PLA, 0.2% sisal fibers, and 4.0% coir fibers, followed closely with a water absorption of 2.70%. These findings suggest that the incorporation of sisal and coir fibers into PLA increases water absorption, thereby increasing the hydrophilicity of the material. This increase can be attributed to the hydroxyl groups (–OH) present in the natural fibers. A comparison of RUN 3 with RUN 1 (95.00% PLA, 4.0% sisal fibers, and 1.0% coir fibers) and RUN 4 with RUN 6 (95.8% PLA, 4.0% sisal fibers, and 0.2% coir fibers) revealed that the coir fibers had a greater impact on water absorption than the sisal fibers. A higher proportion of coir fibers led to increased water absorption. It was also evident that the water absorption of all composites was relatively low during the first 24 h, as moisture initially penetrated the outer PLA surface [40,41,42]. Over time, as moisture reached the porous and hydrophilic fibers, water absorption increased rapidly until equilibrium was reached. Azevedo et al. reported that water absorption follows the Fickian diffusion model [43]. The degree of water absorption is influenced by the amount of reinforced fibers, which affect the void region. As illustrated in Figure 10, a higher number of sisal or coir fibers resulted in increased water absorption. This absorption led to the hydrolytic degradation of PLA, reducing its molecular weight and mechanical strength, which caused embrittlement and a decline in the tensile strength and stiffness.

### 3.9. Morphology of the Hybrid Composites

**Figure 11:**
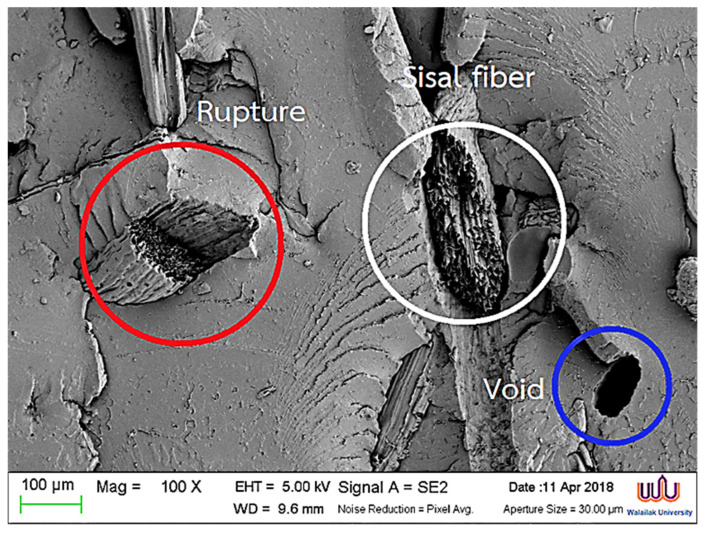
Morphology of the fracture area of the hybrid composites.

The morphology of the fracture area of the hybrid composites, as observed via SEM, is presented in Figure 11. During impact testing, the fibers exhibited tearing, with red-circled areas highlighting the detachment between the PLA matrix and the reinforcing fibers, leading to fiber breakage. This implies that the composite materials reinforced with sisal and coir fibers possessed enhanced mechanical properties due to effective stress transfer from the matrix to the reinforcing fibers [44,45,46]. Additionally, fiber detachment was noted under impact, which was attributed to incomplete adhesion between the PLA matrix and the fibers, resulting in the formation of voids, as observed in the blue-circled areas. Moreover, the moisture content during processing led to issues such as voids, poor fiber wetting, and incomplete curing, ultimately compromising the final product’s quality.

**Figure 12:**
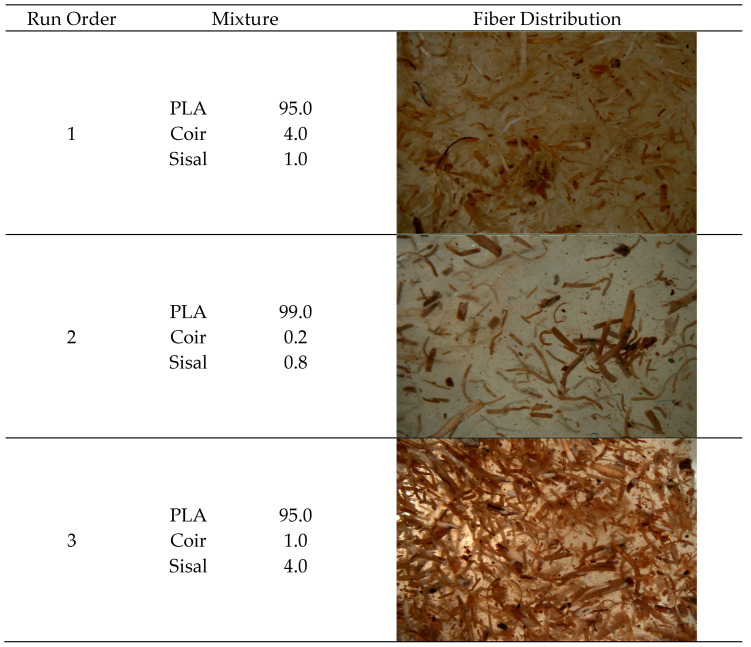

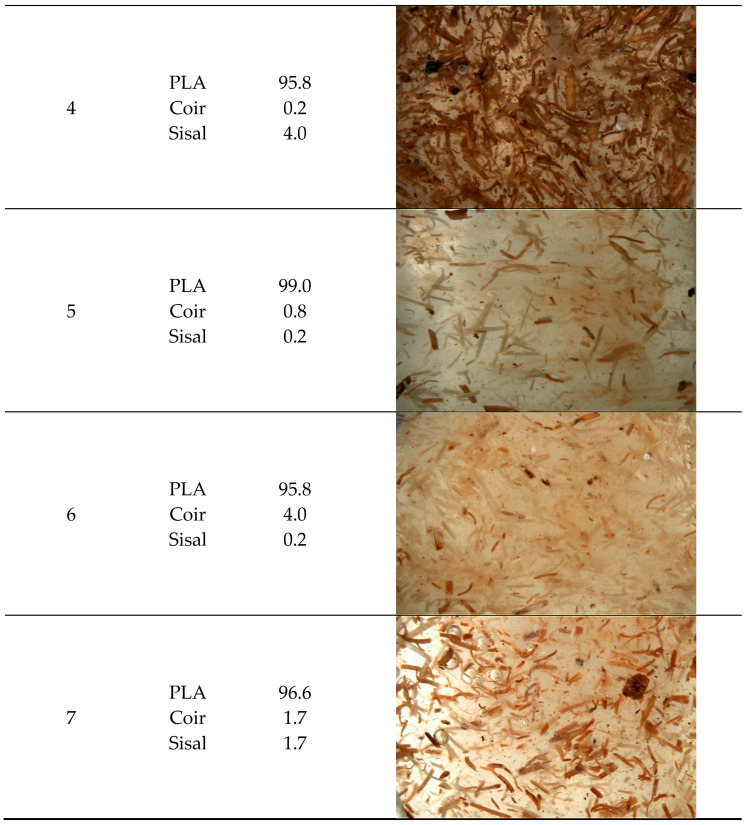
Sisal and coir fiber distribution.

Composite images were recorded using an optical microscope at 500× magnification, as shown in Figure 12. These images displayed high resolution, uniform distribution, and sufficient contrast to clearly distinguish the key features of the sample, including the PLA and fibers.

## 4. Conclusions

Hybrid composites were developed using a constrained mixture design, a statistical tool employed for optimizing the proportions of components to achieve the desirable composite properties. The results showed that combining PLA, sisal, and coir enhanced the properties of the composites. The highest tensile strength of 58.09 MPa was obtained with a PLA:sisal:coir volume ratio of 98.58:1.22:0.20, while the highest impact strength of 30.14 kJ/m^2^ was obtained with a ratio of 95.00:1.08:3.92. Compared to PLA, the sisal and coir fibers had the least influence on the tensile strength; however, they significantly enhanced the impact strength of the hybrid composites. The experimentally optimized composition ratio for the components was PLA:sisal:coir = 95.22:0.78:4.0, which achieved a tensile strength of 51.08 MPa and an impact strength of 26.59 kJ/m^2^, surpassing the values of pure PLA. Notably, the reinforcement with sisal and coir fibers improved PLA’s properties without any detrimental effects. Morphological analysis revealed that the hybrid composite exhibited superior mechanical properties than PLA. These enhancements are attributed to the inherent properties of the fibers, their interaction with the PLA matrix, and the processing conditions. Sisal fibers, characterized by their higher aspect ratio and smoother surface, led to composites with a higher tensile strength and stiffness but required surface treatments to enhance fiber–matrix adhesion. In contrast, coir fibers, with their rougher texture and higher lignin content, provided better mechanical interlocking but resulted in more void formation and less uniform dispersion. Effective optimization of fiber treatment, dispersion, and orientation is key for achieving the desired properties in natural fiber-reinforced PLA composites.

Furthermore, the hybrid PLA composites exhibited good mechanical properties and sustainability, making them suitable for lightweight applications such as automotive dashboards, wall panels, bulletproof vests, and so on. While the natural hydrophilicity of these fibers poses challenges under moist conditions, advancements in surface treatments such as chemical modifications or hybrid composite designs could mitigate moisture absorption, preserving fiber–matrix integrity and enhancing mechanical performance in demanding environments.

## Figures and Tables

**Table 1:** Physical properties of PLA.

Physical Properties		ASTM Method
Specific Gravity, g/cc	1.24	D792 [27]
Relative Viscosity	4.0	D5225 [28]
Peak Melt Temperature, °C	145–160	D3418 [29]
Glass Transition Temperature, °C	55–60	D3418 [29]
Clarity	Transparent	-
Melt Temperature, °C	210	-
Mechanical Property		
Tensile Yield Strength, MPa	60	D882 [30]
Tensile Strength at Break, MPa	53	D882 [30]
Tensile Modulus, GPa	3.6	D882 [30]
Tensile Elongation, %	6	D882 [30]
Notched Izod Impact, J/m	16	D256 [31]
Flexural Strength, MPa	83	D790 [32]
Flexural Modulus, MPa	3.8	D790 [32]

**Table 2:** Component bounds specified in amount.

Component	Lower	Upper	Constraints
PLA (X_1_)	95	99	95 ≤ X_1_ ≤ 99
Sisal (X_2_)	0.2	4	X_2_ + X_3_ ≤0.5
Coir (X_3_)	0.2	4	

**Table 3:** Diameter of sisal and coir fibers.

Fiber	Dia. (µm)
Sisal	127 ± 30
Coir	213 ± 56

**Table 4:** Density of sisal and coir.

Fiber	Density (g/cm^3^)
Sisal	127 ± 30
Coir	213 ± 56

**Table 5:** Experimental runs of mixture design and response and tensile and impact strengths.

Run Order	PLA	Sisal	Coir	Tensile Strength (MPa)	Impact Strength (kJ/m^2^)
1	95.0	4.0	1.0	43.99 ± 5.52	24.69 ± 4.18
2	99.0	0.2	0.8	57.24 ± 1.55	4.79 ± 0.64
3	95.0	1.0	4.0	51.10 ± 4.07	29.96 ± 6.15
4	95.8	0.2	4.0	52.16 ± 3.99	17.65 ± 1.59
5	99.0	0.8	0.2	56.74 ± 2.55	8.32 ± 3.19
6	95.8	4.0	0.2	50.97 ± 2.09	15.52 ± 1.22
7	96.6	1.7	1.7	52.87 ± 2.10	11.83 ± 3.47

**Table 6:** ANOVA for tensile strength.

Source	DF	Adj SS	Adj MS	*p* Value
Regression	4	348.73	87.18	<0.001
Linear	2	63.23	31.62	<0.001
Quadratic	2	52.36	26.18	<0.001

**Table 7:** ANOVA for impact strength.

Source	DF	Adj SS	Adj MS	*p* Value
Regression	5	1425.55	285.11	0.000
Linear	2	193.36	96.68	0.000
Quadratic	3	255.49	85.16	0.000

**Table 8:** Experimental runs of mixture design and response, elongation, and modulus.

Run Order	PLA, X_1_ (%)	Sisal, X_2_ (%)	Coir, X_3_ (%)	Elongation at Break (%)	Young’s Modulus (GPa)
PLA	100	0	0	2.06 ± 0.07	19.95 ± 0.45
1	95.0	4.0	1.0	2.59 ± 0.04	16.97 ± 0.89
2	99.0	0.2	0.8	3.51 ± 0.09	16.31 ± 0.45
3	95.0	1.0	4.0	2.87 ± 0.06	17.84 ± 0.86
4	95.8	0.2	4.0	2.81 ± 0.07	18.57 ± 0.58
5	99.0	0.8	0.2	3.55 ± 0.06	15.98 ± 0.41
6	95.8	4.0	0.2	3.36 ± 0.12	15.17 ± 0.73
7	96.6	1.7	1.7	3.37 ± 0.07	15.70 ± 0.38

**Table 9:** Results of hypothesis testing.

One-Sample T-Test	Tensile Strength	Impact Strength
Mean	52.02	25.60
Standard Deviation	1.431	1.006
95% CI	(50.43, 53.98)	(24.35, 26.85)
T	1.75	−2.21
*p* Value	0.154	0.092

## Data Availability

The original contributions presented in the study are included in the article, further inquiries can be directed to the corresponding author.
